# Immunohistochemical expression of Skp2 protein in oral nevi and melanoma

**DOI:** 10.4317/medoral.18781

**Published:** 2013-02-05

**Authors:** Bruno A. Benevenuto-de-Andrade, Jorge E. León, Román Carlos, Wilson Delgado-Azañero, Adalberto Mosqueda-Taylor, Oslei Paes-de-Almeida

**Affiliations:** 1 DDS, MSc. Oral Pathology Section, Department of Oral Diagnosis, Piracicaba Dental School, University of Campinas (UNICAMP), Piracicaba, São Paulo, Brazil; 2 DDS, PhD. Department of Morphology, Stomatology and Oral Pathology, Dentistry School, University of São Paulo (USP), Ribeirão Preto, São Paulo, Brazil; 3 DDS. Centro Clínico de Cabeza y Cuello, Ciudad de Guatemala, Guatemala; 4 DDS, PhD. Departamento de Patología, Medicina y Cirugía Oral. Facultad de Estomatología. Universidad Peruana Cayetano Heredia, Lima, Perú; 5 DDS, MSc. Departamento de Atención a la Salud. Universidad Autónoma Metropolitana Xochimilco, México, D.F; 6 DDS, PhD. Oral Pathology Section, Department of Oral Diagnosis, Piracicaba Dental School, University of Campinas (UNICAMP), Piracicaba, São Paulo, Brazil

## Abstract

Objective: The aim of this study was to analyze the immunohistochemical expression of Skp2 protein in 38 oral nevi and 11 primary oral melanomas. Study Design: Expression of this ubiquitin protein was evaluated by immunohistochemistry in 49 oral melanocytic lesions, including 38 intramucosal nevi and 11 primary oral melanomas. The labeling index (LI) was assessed considering the percentage of cells expressing nuclear positivity out of the total number of cells, counting 1000 cells per slide. Results: Skp2 protein was rarely expressed in intramucosal nevi, in contrast to oral melanomas, which showed high levels of this protein. Conclusion: These results indicate that Skp2 protein may play a role in the development and progression of oral melanomas, and it also could be useful as an immunohistochemical marker for differential diagnosis of oral benign and malignant melanocytic lesions.

** Key words:**Oral melanoma, oral nevi, Skp2, cell cycle, immunohistochemistry.

## Introduction

The transformation of melanocytes to melanoma cells involves abnormal cell proliferation associated with alterations in the cell cycle regulatory mechanisms (1). Cell cycle progression requires the coordinated performance of a series of regulating molecules that orchestrate cycle transitions through either mitogenic or antiproliferative signals (2). Disruption of the mechanisms involved in protein synthesis and degradation can lead to abnormal cell proliferation and oncogenesis (3). It is well known that the ubiquitin proteasomal pathway plays a paramount role in the degradation of short-lived regulatory proteins involved in the cell cycle (2).

The ubiquitin ligase complex formed by Skp2, Skp1 and cullin F-box (SCFSKP2) is required for direct ubiquination and proteolysis of p27 and other cell cycle regulatory proteins such as cyclin E and the transcription factor E2F-1, performing an S phase promoting function (4,5). Overexpression of Skp2 results in cell cycle progression and eventually neoplastic transformation, as its levels correlates with histologic grade, clinical aggressiveness and prognosis in lymphomas, oral squamous cell carcinoma, prostate adenocarcinoma, ovarian adenocarcinoma, soft tissue sarcomas, gastric carcinomas and breast cancers (6-8).

Recent studies have shown that Skp2 protein expression is implicated in cutaneous melanoma progression, and it may also serve as a biomarker to detect pre-malignant and malignant lesions (3,9). The immunohistochemical expression of Skp2 protein has not yet been studied in oral benign and malignant melanocytic lesions, and therefore, this is the objective of this study. 

## Material and Methods

Formalin-fixed, paraffin-embedded tissue blocks were obtained from 49 oral melanocytic lesions, corresponding to 38 intramucosal nevi and 11 primary oral melanomas. Intramucosal nevi patients included 30 women and 8 men, aged 16 to 67 years, 13 located in hard palate, 11 in the buccal mucosa, 10 in the gingiva, and 4 the site was not specified. Cases of primary oral melanomas corresponded to 8 women and 3 men, aged 23 to 86 years, 6 located in hard palate, 3 involving the hard palate and upper gingiva, and 2 the upper gingiva. Diagnosis of melanoma was confirmed by clinical and histological characteristics, excluding the presence of melanoma at other anatomical sites and consequently the possibility of oral metastasis. Melanomas were histologically classified according to Prasad et al. (10) and all cases corresponded to level III (very deep invasion).

For immunohistochemical staining, 3 µm thick sections mounted on silane-coated glass slides were used. Briefly, the sections were deparaffinized and rehydrated in graded ethanol solutions. After antigen retrieval with EDTA/Tris buffer (pH 9.0) in a microwave oven (1380 W; Panasonic, São Paulo, Brazil), endogenous peroxidase activity was blocked with 20% H2O2 using five cycles of 5 minutes each. Overnight incubation with the primary antibody Skp2 (Santa Cruz Biotechnology, Santa Cruz, California USA) diluted in BSA (bovine serum albumin-1:200) was followed by incubation with the secondary antibody conjugated with polymer dextran marked with peroxidase (Dako EnVision Labeled Polymer; Dako, Glostrup, Denmark). Reactions were developed with a solution containing 0.6 mg/ml 3,3-diaminobenzidine tetrahydrochloride (DAB, Sigma, St. Louis, MO, USA) and 0.01% H2O2 and counterstained with Carazzi’s hematoxylin. Positive and negative controls were included in all reactions. Only nuclear staining was considered positive. As we used DAB as the developer, the reactions could be confounded by melanin in the cytoplasm of benign and malignant melanocytes, but this was not a problem for Skp2 because it is a nuclear marker.

The labeling index (LI) was assessed considering the percentage of cells expressing nuclear positivity out of the total number of cells, counting 1000 cells per slide. The slides were examined under a Leica DMR microscope and images were captured using a Leica digital camera (Leica Microsystems Inc., 1700 Leider Lane, Buffalo Grove, IL, USA). Immunoreactive cells were counted randomly with a minimum of 10 high-power fields (x400), with the help of an image computer analyzer (ImageJ, Image Pro-cessing and Analysis in Java).

## Results

**Figure 1:**
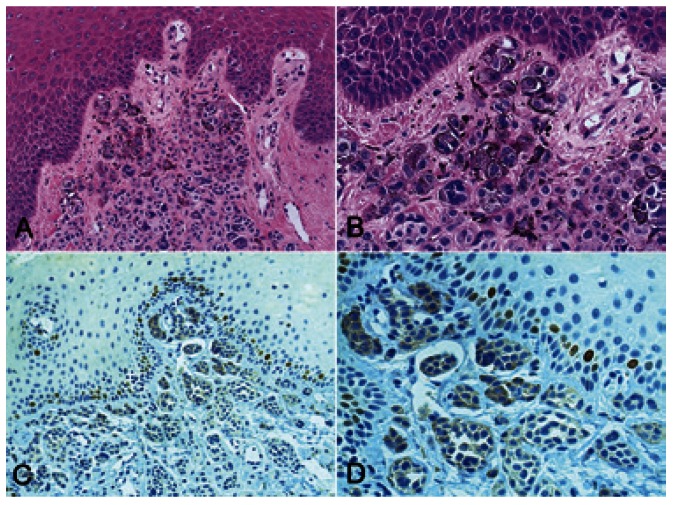
Intramucosal nevi of the gingiva showing sheets and nests of pigmented nevomelanocytes spreading into the underlying connective tissue (H&E, A- x200, B- x400). Intramucosal nevi showing by immunohistochemistry nuclear Skp2 protein expression only in basal and suprabasal cells of the normal oral epithelium, serving as an internal positive control, while the nevic cells are negative (immunoperoxidase, C- x200, D- x400).

**Figure 2:**
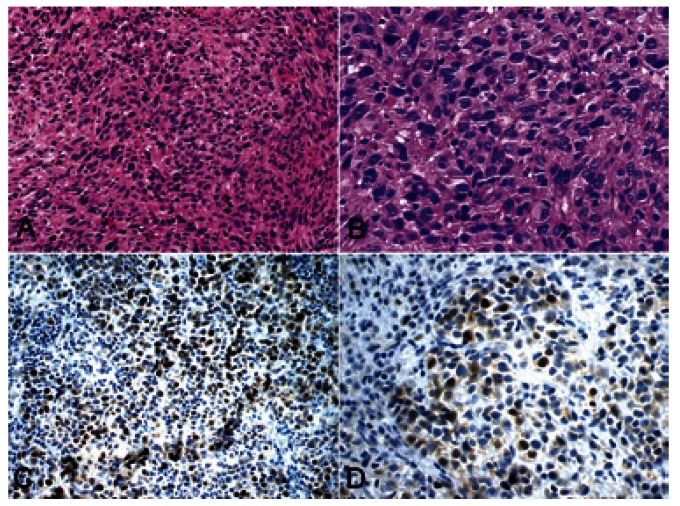
Oral melanoma of the hard palate showing pleomorphic epithelioid neoplastic cells arranged in solid pattern (H&E, A- x200, B- x400). Nuclear expression by immunohistochemistry of Skp2 protein in the tumor cells of oral melanoma (immunoperoxidase, C- x200, D- x400).

The epithelial cells of the normal oral mucosa in nevi and melanomas showed Skp2 protein positivity restricted to the nuclei of basal epithelial cells, together with some cells in the immediate suprabasal layers, serving as an internal positive control. The most superficial cells of the epithelium were negative in all cases (Fig. 1). Skp2 protein was rarely expressed in intramucosal nevi, with LI lower than 1% in all cases. The location of Skp2 expression was heterogeneous, not involving specific areas of nevus cells nests (Fig. 1). In primary oral melanomas, 17.5% (range: 8.7% to 36.5%) of malignant cells expressed Skp2 in the nucleus, mainly on the superficial central compartment of the tumor rather than in the tumor margins (Fig. 2).

## Discussion

Melanoma is known to exhibit aberrant expression of cell-cycle-regulating proteins. The F-box protein Skp2 is a component of the ubiquitin protein ligases that play a critical role in the regulation of G to S phase progression (6). Cell cycle progression is driven by an increase of Skp2, which is responsible for ubiquination of some cell cycle proteins such as cyclin E and p27 (4,5). In this study we evaluated the immunohistochemical expression of Skp2 protein in 38 intramucosal nevi and 11 primary oral melanomas, as there are no data on immunohistochemical expression of Skp2 in oral melanocytic lesions and only 4 reports consider its relevance in cutaneous melanocytic lesions (3,9,11,12).

We found low expression of nuclear Skp2 in oral nevi compared to melanomas, confirming an oncogenic potential of Skp2. This is also in accordance with literature reports of negative or weak expression of Skp2 protein in benign cutaneous melanocytic lesions and high levels in melanomas (3,9,11-13). Li et al. (3) reported a progressive and significant increase in the nuclear expression of Skp2, moving from melanocytic nevi to melanoma in situ, primary cutaneous melanoma and metastatic melanoma respectively, suggesting that this protein is implicated in melanoma progression. High levels of Skp2 have also been shown in a variety of cancers such as prostate (14), oral squamous carcinoma (7,15) and colorectal carcinomas (16). Also attesting the importance of Skp2 in tumor development, there is a report using transgenic mouse model where Skp2 overexpression induced prostatic hyperplasia, dysplasia, and low-grade carcinoma (17).

The present study did not consider the relation between expression levels of Skp2 protein with stage and prognosis of oral melanomas, but it is well known that oral melanomas have a poor prognosis and all our cases showed deep invasion. Nevertheless, it is has been shown that Skp2 nuclear protein expression have prognostic impact in some human cancers such as squamous cell carcinoma (7). Nevertheless, in cutaneous melanomas results are controversial, as increased expression of nuclear Skp2 have been correlated either with reduced survival time (9), or that it was not associated with prognosis (3).

In conclusion, our data suggest that increased Skp2 expression plays a role in the development of oral melanomas. Overexpression of Skp2 may represent an important mechanism in abrogating the cell-cycle inhibitory and apoptotic effects of some cell cycle proteins such as of p27 and cyclin E, increasing their degradation, thus contributing to the expansion of malignant clones of melanocytes and tumor progression. Besides to be involved in the pathogenesis of oral melanomas, Skp2 could also be useful as an additional diagnostic marker for differential diagnosis of oral benign and malignant melanocytic lesions.
